# Supplementary material for: Genome-Wide Identification of the OPT Gene Family and Screening of Sb-Responsive Genes in Brassica juncea
Source: Plants (Basel). 2025 Nov 6;14(21):3399. doi: 10.3390/plants14213399 (PMC12609508; doi:10.3390/plants14213399)
Supplement: Supplementary file 1 [file plants-14-03399-s001.zip › Supplementary Figures.pdf]

Supplementary Figures S1-S5

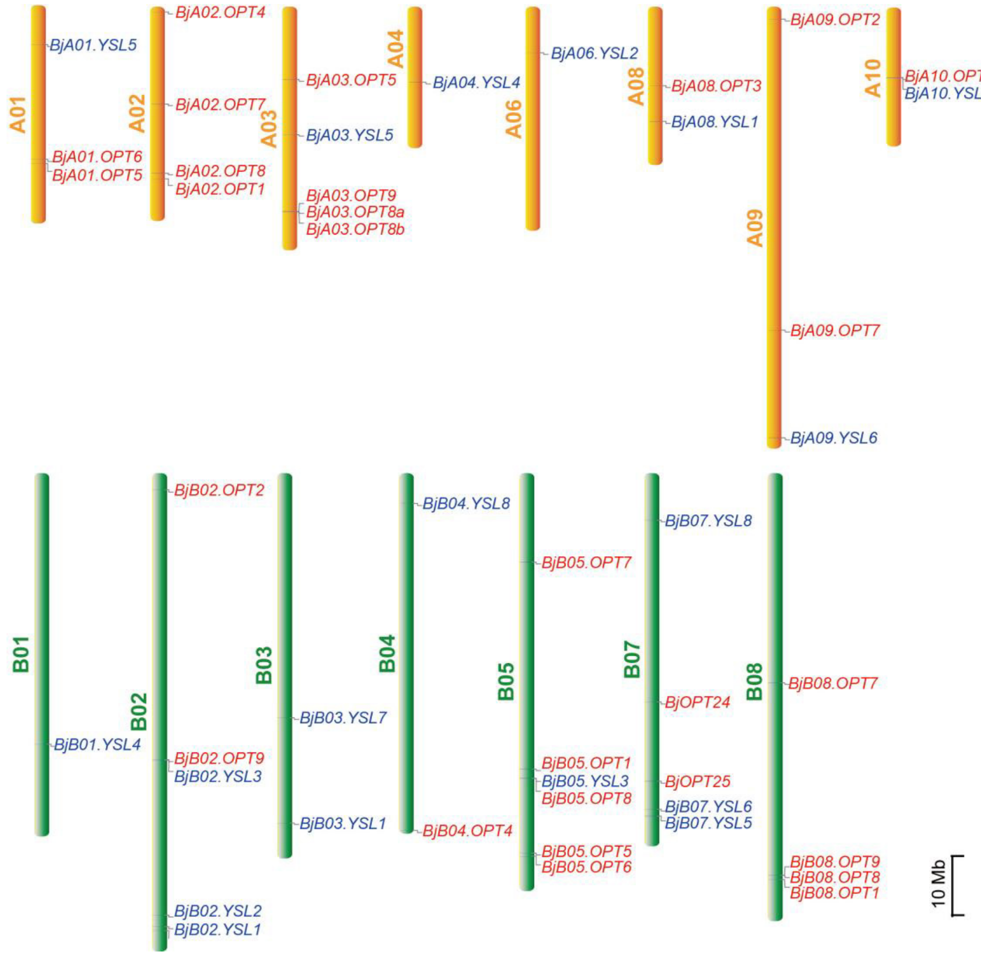

Figure S1. Chromosomal Localization Analysis of BjOPT Genes. Red fonts indicate BjOPT members; blue fonts denote BjYSL members. Scale bar: 10 Mb.

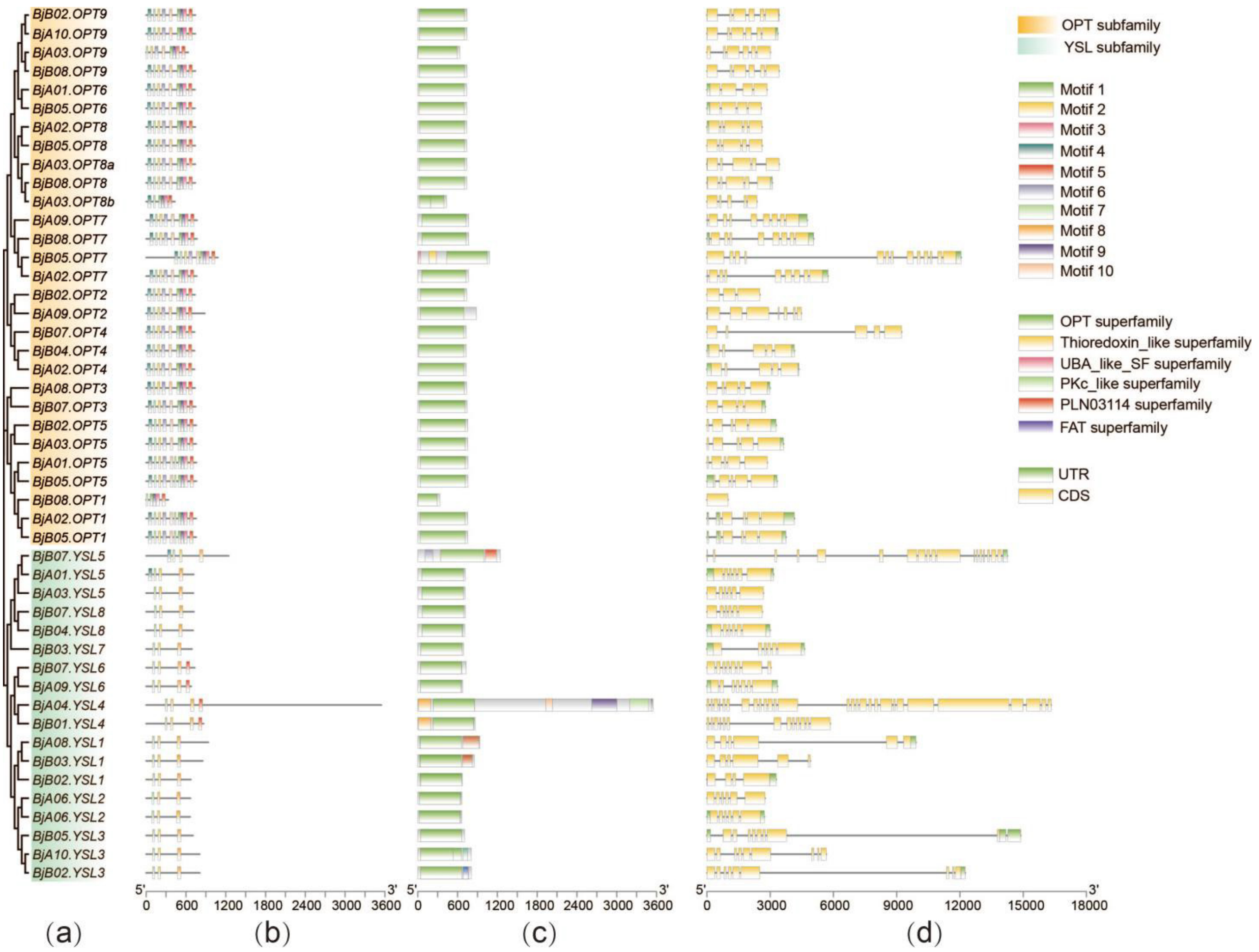

Figure S2. Phylogenetic relationship, conserved motif, protein domain and gene structure analyses of BjOPT proteins. (a) Phylogenetic tree; (b) Conserved motif; (c) Protein domain; (d) Gene structure

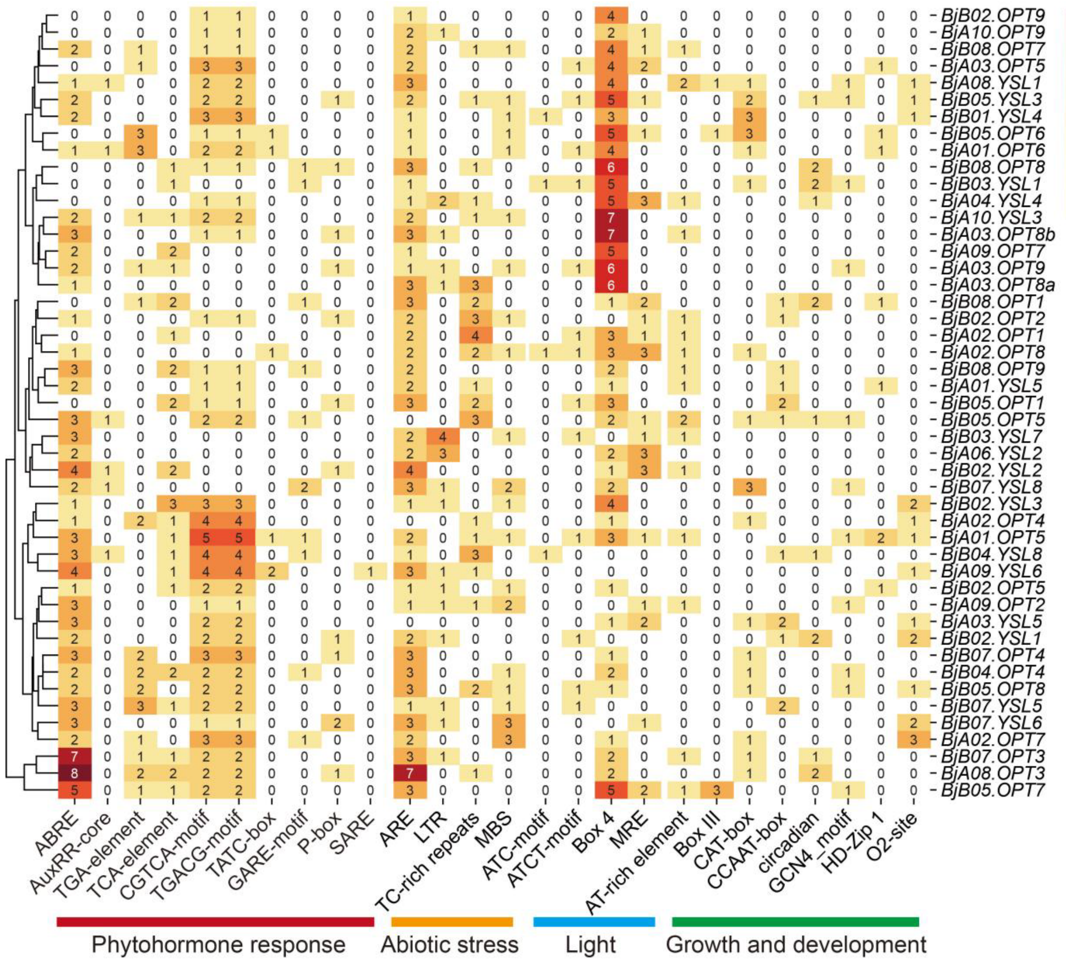

Figure S3. Heatmap of Cis-Regulatory element distribution in *BjOPT* promoters. Numbers indicate the count of specific cis-acting elements.

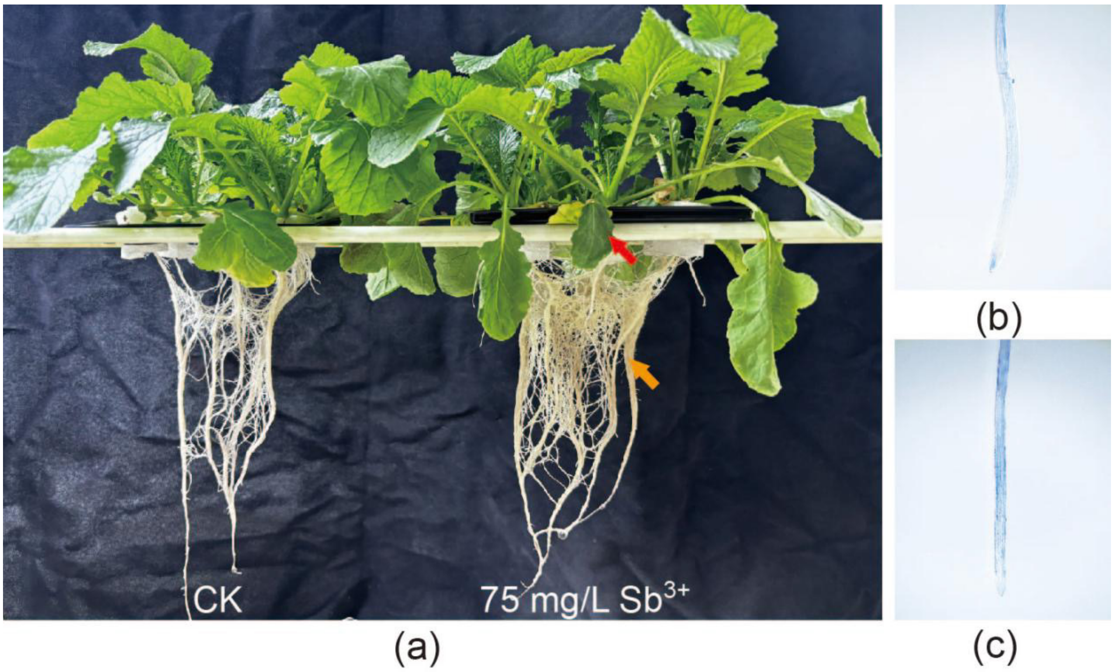

Figure S4. Phenotypic response of *B. juncea* seedlings to Sb stress. (a) Whole-seedling phenotype following 24 h of Sb exposure. Red and orange arrows point to leaf and root tissues, respectively, that were altered by Sb exposure; (b, c) Evans Blue staining of root tissues from control (b) and Sb-treated (c) seedlings.

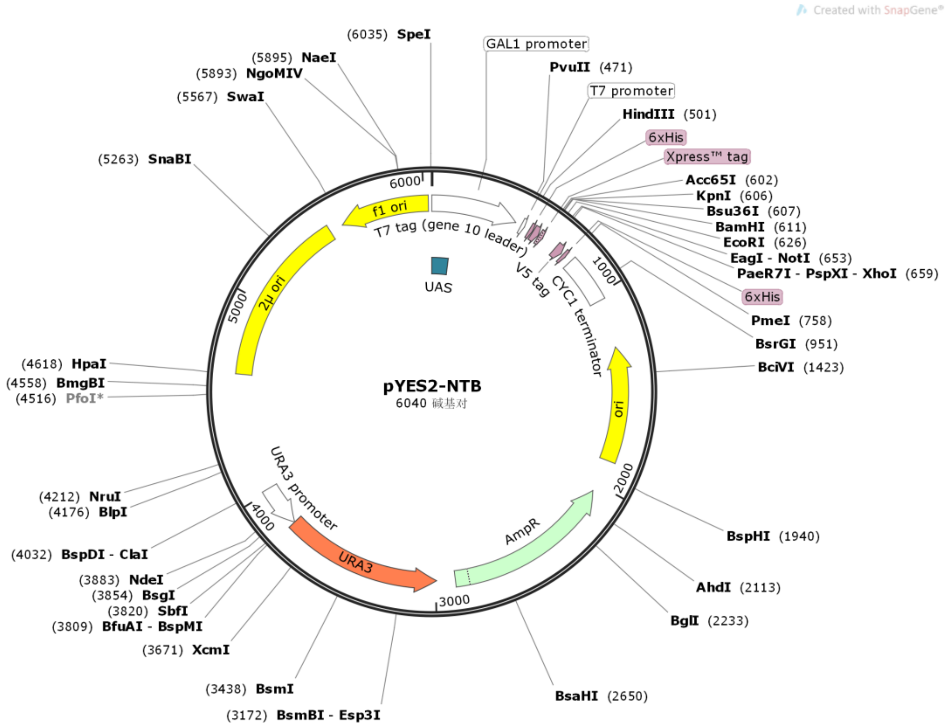

Figure S5. Schematic diagram of the pYES2-NTB plasmid used in this study.

.
